# Decoding dental images: a comprehensive review of fractal analysis

**DOI:** 10.1038/s41405-025-00365-1

**Published:** 2025-08-21

**Authors:** Lisamarie Shalini Linhares Colaco, Yogesh Chhaparwal, Vathsala Patil, Komal Smriti

**Affiliations:** Department of Oral Medicine and Radiology, Manipal College of Dental Sciences, Manipal, Manipal Academy of Higher Education, Manipal, Karnataka India

**Keywords:** Digital radiography in dentistry, Oral diseases

## Abstract

**Objectives:**

New tools aid in the diagnosis of diseases and thus help in advancing patient care. “Fractal Analysis” is a versatile method of applying nontraditional mathematics to patterns that are beyond understanding with traditional Euclidean concepts. This analysis can be used on radiographic and non-radiographic images in dentistry. In this review we aim to identify the usefulness of fractal analysis in dentistry in radiographic images, its applications and future scope.

**Materials and Methods:**

Articles published between 1992 and 2024 were retrieved through an electronic search of Medline via PubMed, Scopus, and Google Scholar databases. The search, which was limited to articles published in English, aimed to identify relevant studies by employing the following keywords: “fractal analysis,” “dental radiographs,” “mandibular,” “panoramic radiographs,” and “radiography.” Ultimately, 76 articles that addressed the application of fractal analysis in dental radiographs were selected.

**Results:**

Fractal analysis can reveal alterations in bone and in images of morphologically altered tissue, however no set values exist which could be used as a standard for diagnosing various conditions.

**Conclusion:**

Fractal Analysis can potentially be used as an adjunct to diagnostic tests as it is shown to identify alterations in bony and trabeculae patterns.

## Introduction

The world is rapidly progressing towards a period of almost complete digitalization, marked by significant transformations across various domains. Technological advancements persist at an accelerated rate, signifying a transformative phase in healthcare developments. Robotics, artificial intelligence, and progress in machinery have significantly enhanced the quality of care available to patients. One such advancement is “Fractal Analysis,” a concept that has existed for over 30 years but is being gradually and steadily implemented in the domains of medicine and dentistry [1]. Fractal analysis (FA) represents an innovative and adaptable approach to employing non-traditional mathematical concepts to patterns that are challenging to comprehend using conventional Euclidean frameworks. This domain was established to characterize computer-generated fractals; however, fractals are not exclusively limited to computer-generated imagery. Instead, Euclidean geometry is evident in commonplace objects such as oranges and watermelons, while fractal geometry is discernible in familiar forms including undulating coastlines, expanding crystals, and spiral galaxies [2, 3]. Within the human body, the most prominent fractal structures are observed in coronary vessels, Purkinje fibers in the heart, the lungs, neurons, trabeculae in bone, and blood vessels in the eye [3]. Benoit Mandelbrot is regarded as the father of contemporary fractal analysis; he disseminated the notion of fractals in his publication, “How Long Is the Coast of Britain? Statistical Self-Similarity and Fractional Dimension” [4], which appeared in 1967. This analysis has been utilized in various applications, including heart rate assessment, diagnostic imaging, cancer research [5], fractal analysis of complex networks, categorization of histopathology slides in medical contexts, evaluation of fractal landscapes or coastline complexity [1], electrical engineering, enzyme/enzymology (particularly in relation to Michaelis-Menten kinetics), the creation of novel music, the production of diverse art forms, signal and image compression, urban development, neuroscience, pathology, geology, geography, archaeology, seismology, soil studies [6], as well as the design of computer and video games, especially in the context of computer graphics for organic environments and as an element of procedural generation [7, 8].

In dental diagnosis, radiographs are an essential adjuvant. To help with precise clinical evaluation, a variety of imaging techniques are frequently used, including intraoral periapical radiographs (IOPA), orthopantomograms (OPG), and cone-beam computed tomography (CBCT). Tools that can objectively identify changes in bone density and trabecular patterns are becoming more and more important as a result of the quick development of dental technology. Particularly in situations involving bone pathology, periodontal disease and systemic diseases affecting the bone. These tools provide invaluable assistance in early diagnosis and treatment planning thereby reducing the load on the radiologist. In this review, we seek to examine the diverse applications of fractal analysis in dentistry using radiographic images and the results of these applications.

## Materials and Methods

This review was based on the question: How common is the application of fractal analysis in dental radiographs and how useful is it? A literature search was performed in PubMed/MEDLINE, Ovid, LILACS, Web of Science and Google Scholar for all the published articles related to fractal analysis and its applications in dentistry. The last search was performed on March 10th, 2024.

**Fig. 1:**
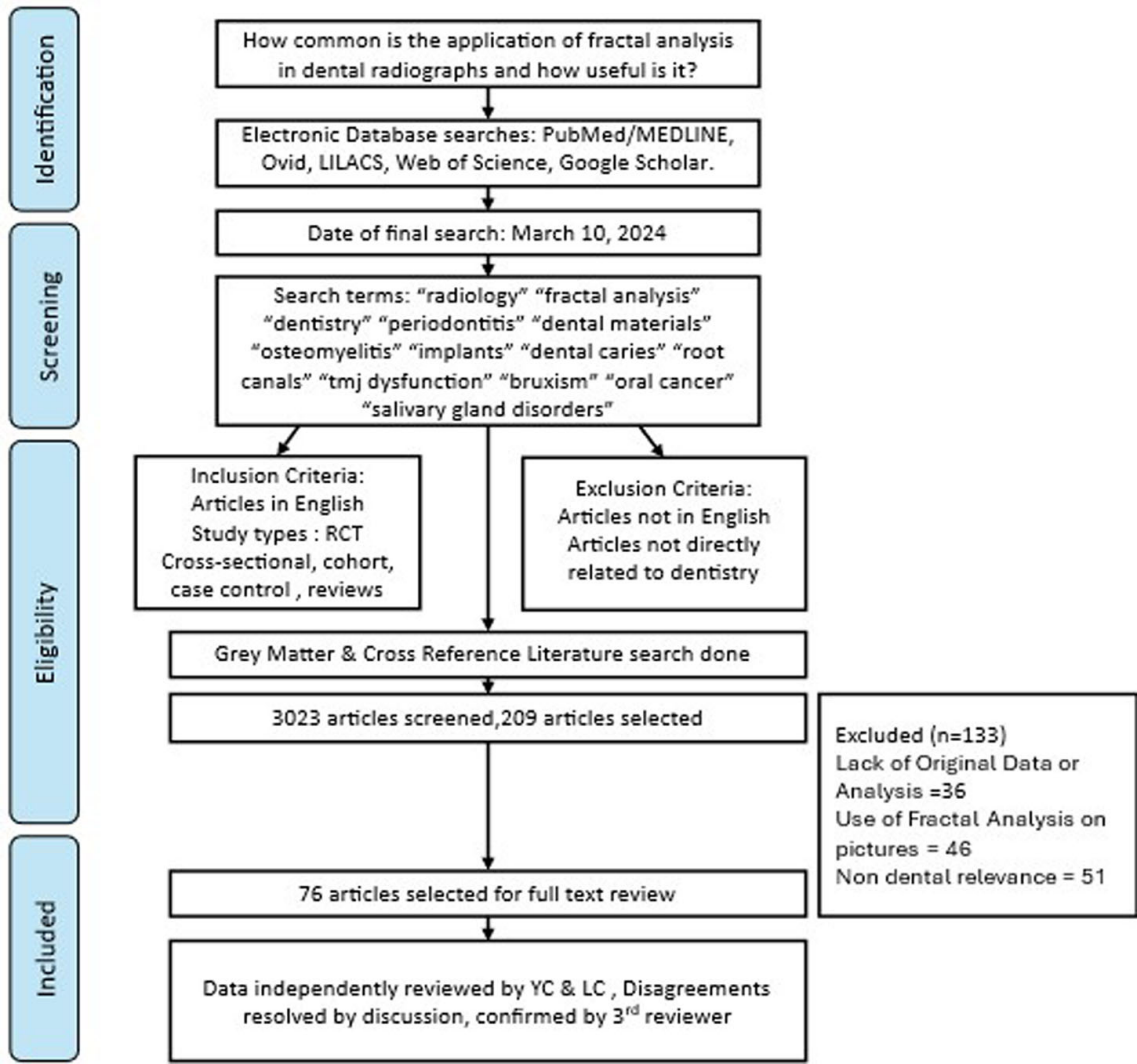
Flowchart for selection process of studies.

The following search terms were used: “radiology” “fractal analysis” “dentistry” “periodontitis” “dental materials” “osteomyelitis” “implants” “dental caries” “root canals” “tmj dysfunction” “bruxism” “oral cancer” “salivary gland disorders”. Publications in English were included. Publications in other languages were excluded. Randomized and nonrandomized trials, cross-sectional studies, cohort, case-control studies, and systematic and literature reviews were included for data collection. The Grey Matters & Google scholar was also searched for relevant articles. The cross-reference of all studies was searched to include anything relevant to the topic. All studies that did not refer to the application of fractal analysis in dentistry were excluded. The total of all the results (3023 articles) was compiled in the Mendeley reference manager (v 2.85.0), and duplicates were removed. After removing the duplicates, 209 articles were included, and 76 articles were included for full-text screening. The data collected were reviewed by all the authors. Any disagreements were mutually discussed between the two reviewers (YC & LC), and a consensus was reached. A 3rd reviewer further reviewed the articles selected for analysis -Fig. 1. All the data related to the applications of fractal analysis in dental radiology were analyzed and reported

## Results and Discussion

**Fig. 2:**
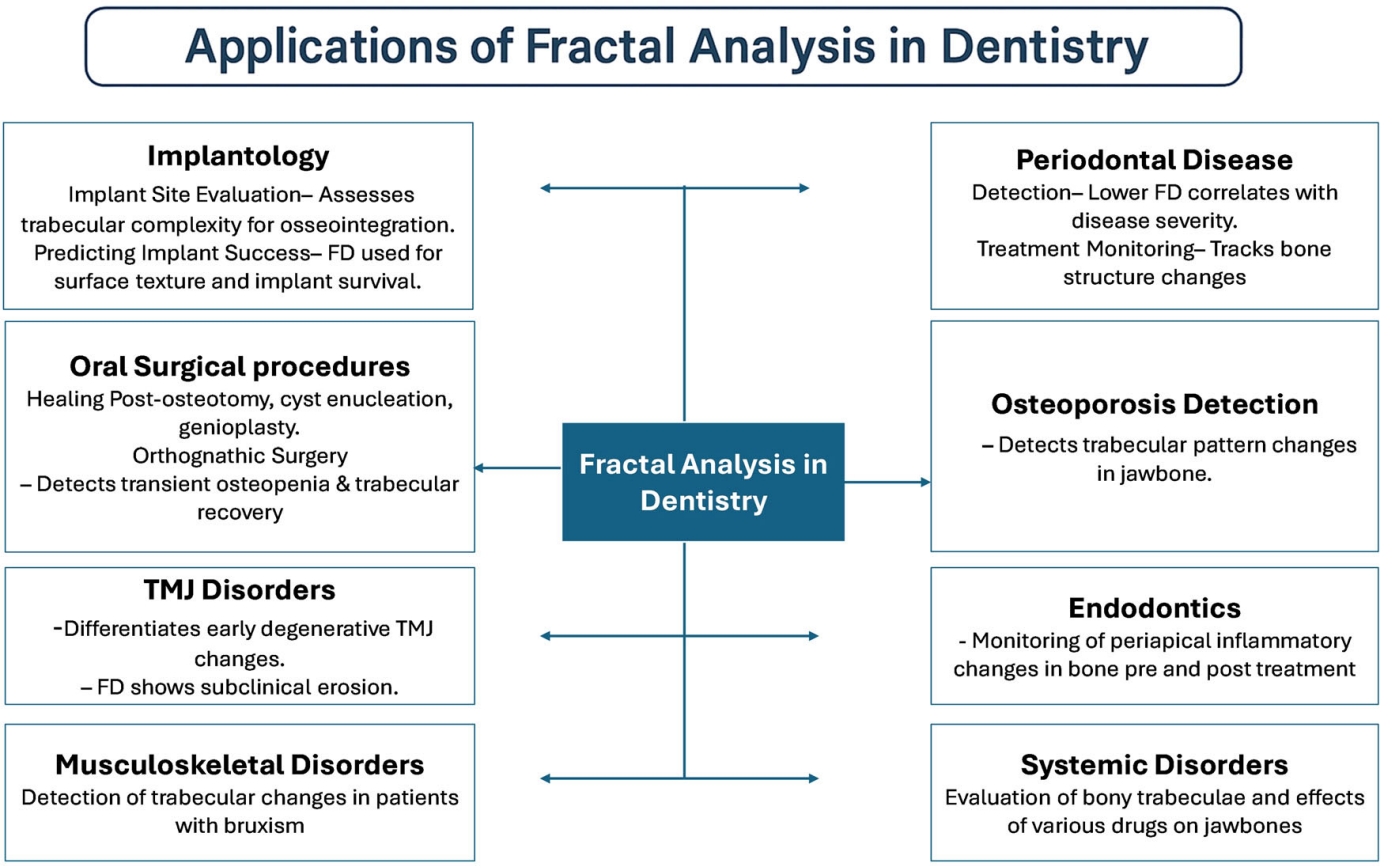
Applications of Fractal Analysis in various dental fields.

The capacity of fractal geometry to quantify and analyze the irregular, fragmented forms of natural objects that are not amenable to measurement by conventional mathematical methods has resulted in the widespread adoption of fractal analysis across various disciplines, particularly in the medical domain, where fractal structures are prevalent [2, 3, 9]. The mandibular bone, characterized by its branching trabeculae, demonstrates statistical self-similarity. Consequently, the utilization of fractal geometry and the quantification of fractal dimensions (FD) can facilitate the assessment of the complexity inherent in the trabecular pattern and bone architecture [10]. Several prevalent applications where FA has demonstrated its utility include quantifying trabecular alterations following surgical and orthodontic interventions, assessing the surface roughness of implants, and evaluating the healing of periapical lesions subsequent to root canal therapy. Pictorial fractal analysis is likewise employed to assess histopathological specimens of premalignant and malignant tissue [9, 11]. Nevertheless, a more prevalent utilization of FA in dentistry involves the assessment of radiographic images to track alterations in the morphological configurations of the jawbones. Numerous investigations have been conducted primarily to examine the trabecular architecture of the mandible through the utilization of intraoral periapical radiographs, panoramic radiographs, and CBCT images. The most prevalent technique in fractal analysis is the box counting method developed by White and Rudolph for the processing of dental images [12]. Figure 2 depicts the applications of fractal analysis in various areas of dentistry.

### Osteoporosis detection

Osteoporosis is a global health issue marked by low bone mass and weakened bone structure, leading to a higher risk of fractures. Dental radiographs, commonly used in dental checkups, help in assessing bone conditions due to their accessibility, affordability, and low radiation exposure, making them an effective screening tool for osteoporosis risk. Research by Southard et al., focused on fractal analysis in osteoporotic subjects [13–16]. They first examined the link between fractal dimensions (FD) in the jawbone and bone density in rabbits with induced osteoporosis, findings showed reduced FD with higher steroid doses but no correlation with spine and mandible density [16]. In healthy women, FD in the jawbone was positively linked to local bone density but not to other areas like the spine [14]. Other studies, including those by Bollen et al. [17], and Park et al. [18], found higher FD values in panoramic and periapical radiographs of osteoporotic patients. Yasar & Akgunlu [19–21] carried out a series of studies, first on digital periapical images and then on panoramic images, they meticulously evaluated if fractal dimensions could be considered to detect osteoporosis and could differentiate the lacunarity and trabecular pattern of the mandible. They found a mean value of FD in osteoporotic subjects as 1.40 and 1.39 in non-osteoporotic subjects. Thereby confirming the usefulness of FD in detection of osteoporotic bone. Sindeaux et al. [22], evaluated 133 panoramic radiographs of 84 females and 49 males and they considered bone mineral density (BMD) values determined by dual-energy X-ray absorptiometry (DXA) as the gold standard. They found lower FD values in mandibular bone among postmenopausal women (normal = 1.420 ± 0.079, osteoporotic= 1.354 ± 0.090) and older men with osteoporosis (normal=1.373 ± 0.069, osteoporotic=1.371 ± 0.059). Their results suggested that cortical bone measurements could help identify patients need for further testing for bone density, however the results showed that FD values were not consistent over various regions. Table 1 summarizes all the studies done using fractal analysis to detect osteoporosis.

**Table 1:** Studies using fractal analysis to detect osteoporosis.

Author(s)	Year	Aim/Objective	Key Findings
Doyle et al. [9]	1992	Detect osteoporosis using FD in dental radiographs	Higher FD in postmenopausal women compared to premenopausal
Ruttimann et al. [69]	1992	Measure FD in mandibular radiographs	Higher FD in postmenopausal women
Law et al. [70]	1996	Compare methods for osteoporosis detection	FD increased in osteoporotic cases
Southard et al. [15]	2000	Study FD in rabbits with induced osteoporosis	Mandibular FD decreased with steroid dose
Southard et al. [14]	2001	Study FD and bone density in healthy women	FD positively correlated with mandibular bone density, not with spine/hip/radius
Bollen et al. [17]	2001	Discriminate osteoporosis using FD in panoramic radiograph	FD distinguished osteoporotic cases; periapical radiographs not useful
Park et al. [18]	2005	Predict osteoporosis using FD in periapical radiograph	FD increased in osteoporotic postmenopausal women
Yasar and Akgunlu et al. [21]	2005	Detect changes in edentulous vs dentate mandibles	FD and lacunarity detected changes
Yasar and Akgunlu et al. [19]	2006	Compare FD in osteoporotic vs normal patients	No significant difference
Kim and Nah et al. [71]	2007	Use FD on panoramic radiographs	FD increased in osteoporotic postmenopausal women
Yasar and Akgunlu et al. [20]	2008	Discern MCI categories with FD and lacunarity	Differentiated type 1 from 2 and 3, not between 2 and 3
Sindeaux et al. [22]	2014	Compare FD and MCW in osteoporosis vs normal	Lower FD and MCW in osteoporotic cortical bone
Franciotti et al. [67]	2021	Meta-analysis of FD for osteoporosis detection	FD not reliable; studies too heterogeneous

### Periodontitis detection

Periodontitis is a chronic inflammatory disease that damages the tissues supporting teeth such as gingiva and bone. Detecting periodontitis involves clinical examination, gingival recession, through probing and radiographic assessment. Traditional radiographs often fail to depict radiographic changes until 30 to 50 percent of the bone is lost [23]. To effectively identify chronic periodontitis in its early stages, thorough examinations and evaluations are necessary. Many studies evaluating fractal dimensions of bone in periodontitis patients show lower FD values compared to healthy subjects or those with gingivitis [23–31]. Although various methodologies were used, these studies suggest that FD might help in diagnosing periodontitis, yet it is not applicable for clinical use and requires further validation. Efforts have also been made to model periodontal disease using various quantitative parameters like plaque levels or hormonal factors, but predicting bone loss remains challenging due to multiple influencing factors [23, 27, 32]. One study by Shrout et al., found that FD values were significantly lower in patients with periodontitis(mean fractal value = 2.049) compared to healthy individuals(mean fractal value = 2.061), confirming its potential as an objective detection method [33]. Other research indicated that FD effectively discriminates between healthy gingiva (1.74 ± 0.083), moderate periodontitis(1.66 ± 0.104) and severe periodontitis(1.64 ± 0.095), supporting its use for monitoring changes in bone structure [24]. Moreover, another study illustrated that FD values decrease as periodontitis stages increase, showing a strong negative correlation between FD and bone loss [30]. These studies showed that FD can serve as a quantitative tool for assessing changes in cancellous bone associated with periodontitis. Table 2 summarizes all the studies done using fractal analysis to detect periodontitis.

**Table 2:** Studies using fractal analysis for periodontitis detection.

Author(s)	Year	Aim/Objective	Key Findings
Shrout et al. [33]	1998	Compare FD in periodontitis vs healthy patients	Lower FD in periodontitis patients
Sang-Yun et al. [25]	2001	Compare FD ratios in furcation vs interdental areas	Significant FD ratio difference in patients with furcation involvement
Wagle et al. [72]	2005	Analyze FD in periodontal ligament in rats under load	Increased FD with mechanical loading
Madan et al. [32]	2007	Evaluate fiber organization with relaxin effect	FD useful in studying orthodontic tooth movement
Updike and Nowzari et al. [24]	2008	Detect trabecular changes in periodontitis	FD distinguished periodontitis from healthy, not mild vs severe
Sener et al. [30]	2015	Detect trabecular changes via FD	FD significantly different between healthy and periodontitis groups
Belgin and Serindere et al. [73]	2020	Compare FD in healthy vs periodontitis patients	Significantly lower FD in periodontitis
Korkmaz et al. [26]	2023	Compare FD in aggressive periodontitis	FA detected significant trabecular differences; useful for predicting susceptibility
Mishra et al. [44]	2023	Stage periodontitis using FD	FD decreased with higher stages; strong negative correlation with % bone loss

^*^Fractal Dimension = FD.

### Root Canal Treatment (RCT) -healing and monitoring

Fractal analysis, particularly fractal dimension (FD), is important in root canal treatment to assess bone healing and treatment success. It provides a numerical value that describes the complexity of trabecular bone architecture, allowing for the quantification of bone regeneration and inflammation. Yu YY et al. [34], studied FD changes in periapical lesions after root canal treatment using digitized images from intraoral radiographs before and after treatment at different time points. They found a significant increase in FD after three months of successful treatment ranging from 1.720 to 1.580. Aydin et al. [35], compared FD changes in healthy individuals and type 2 diabetes mellitus patients after root canal treatment. They found increased FD values one-year post-treatment in both groups, with a more significant increase in the healthy group (0.274 ± 0.082) than in the group with diabetes mellitus (0.180 ± 0.114). Both these studies evaluated periapical radiographs. However,Yen YY et al carried out mathematical morphology analysis that increased the specificity of the fractal values. Tosun et al. [36], examined the changes in FD in patients undergoing nonsurgical endodontic retreatment. Increased FD was noted in healed cases (baseline=1.191 ± 0.022; postop=1.308 ± 0.094), while it decreased in unhealed cases (baseline=1.201 ± 0.023; postop=1.148 ± 0.078). These studies showed that changes in the FD values can depict pre and post treatment bony changes. Table 3 summarizes various studies done using fractal analysis for healing and treatment monitoring of bone after a root canal.

**Table 3:** Studies using fractal analysis for evaluating healing and monitoring bone after a root canal.

Author(s)	Year	Aim/Objective	Key Findings
Lee et al. [74]	2005	Evaluate bony changes in apical lesions	FD difference decreased over time post-treatment
Chen et al. [34]	2009	Monitor periapical lesion healing post-RCT	FD increased after successful RCT
Yu et al. [34]	2009	Assess FD pre- and post-RCT	FD lower after 6 months post-RCT
Aydin et al. [35]	2021	Compare FD in diabetic vs. healthy post-RCT	FD increased; lower increase in diabetic patients
Tosun et al. [36]	2022	Compare FD and PAI in retreatment	FD increased in healed cases; FD decreased in unhealed; no strong correlation with PAI

### Implant evaluation

Bone quality is crucial for achieving osseointegration, which determines the stability and success of a dental implant. Proper bone regeneration around the implant ensures long-term load-bearing capacity and functional integration. Wilding et al. [37], studied fractal analysis to track bone healing after dental implants in 18 patients. They found that the bone structure changed significantly around the implant. Traini et al. [38], used fractal analysis to examine how the distance between implants affected blood vessel organization in bone, concluding that a 3 mm distance was better for vascular density than a 2 mm distance. Grizon et al. [39], utilized FD to assess the surface characteristics of titanium implants, suggesting that fractal analysis can evaluate mechanical compatibility. Yi et al. [40], introduced a method to assess bone mechanical properties using FD values from radiographs taken at different angles to create a map of the trabecular structure’s anisotropy. Mundim et al. [41], used periapical radiographs to analyze the bone texture in dental implant planning. They stated that fractal analysis could reliably predict implant stability. Lang et al. [42], researched whether fractal analysis could differentiate between healthy and diseased peri-implant bone and found contrasting results where he stated FD was not a valid distinguishing method. Cansu Kis et al. [43], evaluated microstructural changes around short implants using fractal analysis, concluding it could help predict implant survival based on the trabecular bone’s structure. Mishra et al. [44], also conducted a scoping review on FD in dental radiographs, they noted that most of the studies were done using periapical radiographs, followed by panoramic radiographs. They mentioned that while cone beam computed tomography (CBCT) provides a more accurate 3D view of the bone structure, fewer studies have utilized it compared to traditional methods. Table 4 summarizes all the studies done using fractal analysis to evaluate implants and implant stability.

**Table 4:** Studies using fractal analysis to evaluate implants and implant stability.

Author(s)	Year	Aim/Objective	Key Findings
Wilding et al. [37]	1995	Monitor alveolar bone regeneration post-implant	FD increased near implant neck over time
Grizon et al. [39]	2002	Study implant surface texture	FD useful to measure surface roughness
Jung et al. [75]	2005	Study bone structure change after implants	FD changed post-implantation
Veltri et al. [76]	2007	Relate FD to damping factor	No correlation found
Yi et al. [77]	2007	Analyze anisotropy in bone	Directional FD indicated anisotropy and bone mechanical properties
Lee et al. [78]	2010	Correlating FD with Implant Stability Quotient (ISQ)	Positive correlation with ISQ
Traini et al. [38]	2010	Evaluate vascularization via FA	3 mm inter-implant distance better for vascular density
Zeytinoglu et al. [79]	2015	Monitor peri-implant bone over time	FD decreased 6 months post-loading
Mundim et al. [41]	2016	Use texture analysis for implant planning	FD useful for non-invasive implant planning
Jodha et al. [80]	2020	Study FD on failed zirconia implants	FD consistent across fracture sites; useful for fracture toughness
Lang et al. [42]	2020	Compare FD in healthy vs diseased implants	FD not valid to distinguish peri-implant health
Kis et al. [43]	2020	Assess FD in short implant survival	FD predicted implant survival
Mishra et al. [44]	2022	Review FD in implant stability	Most studies used IOPAR, CBCT, which yielded different results

### Treatment and healing monitoring after surgery

Monitoring trabecular pattern of the jawbones after any surgery is crucial to assess bone healing, detect early signs of infection or complications, and ensure proper integration of grafts, miniplates or implants. Heo et al. [45], and Park et al. [46], studied the fractal dimension (FD) of binary images from panoramic radiographs taken before and after orthognathic surgery for mandibular prognathism. They found their method of FD analysis to be more effective than visual inspection in assessing bone healing. Trabecular changes in the bone post cyst enucleation surgeries in the jaw, transient osteopenia from rapid orthodontic movement, genioplasty surgeries and post osteotomy surgery, could also be evaluated using FD analysis [47–50]. Akbulut et al. [51], evaluated the effectiveness of fractal analysis from hand-wrist radiographs in deciding between conventional or surgery-assisted rapid palatal expansion, suggesting it could be a useful predictor. Table 4 summarizes all the studies done using fractal analysis for healing and treatment monitoring of bone after surgery.

### Fractal analysis of bone pattern in thyroid disorders

S Ergun et al. [52], discussed a 65-year-old patient diagnosed with primary hyperparathyroidism (HPT) due to dental issues. The study reviewed the patient’s medical records and panoramic films from 1997 to 2008, using fractal analysis to assess bone metabolism. The patient showed osteoporotic bone characteristics until a parathyroidectomy, after which biochemical levels normalized, and bone quality improved. The study suggests that fractal dimension (FD) analysis can effectively examine alveolar bone quality in HPT. Ozturk et al. [53], investigated bone changes in the mandible caused by hyperthyroidism and hypothyroidism using fractal analysis on panoramic radiographs. Their findings indicated that hyperthyroid patients had lower FD values than hypothyroid patients, confirming that fractal analysis is useful for early detection of bone density changes related to thyroid disorders, although trabecular regions were affected despite intact mandibular cortical bone.

### Fractal analysis of bone in Rheumatoid arthritis and bruxism

Turkmenoglu et al. [54], compared the FD of mandibular condyles in rheumatoid arthritis (RA) patients to those without RA. They found no significant correlations between FD and bone density in the femoral neck or lumbar spine. However, the fractal analysis was effective in distinguishing RA patients from healthy individuals, even with normal bone density readings. Balkan et al. [55], examined the impact of bruxism on the mandibular bone structure after botulinum toxin-A (BTX-A) injections in patients’ masseter muscles, using fractal analysis. They found that hyperactivity in the masseter muscle increased bone density, whereas BTX-A injections reduced muscle activity and changed bone structure, reflected in decreased FD values.

### Fractal analysis in diagnosing temporomandibular joint disorders

Early identification of degenerative changes in the temporomandibular joint is a challenging task. Role of FA in this aspect has been evaluated by various researchers [50, 56–58]. Canger et al. [56], retrospectively analyzed mandibular condyle in patients with ankylosing spondylitis on panoramic radiographs, they found FD values to be lower in the case group compared to healthy controls ranging from 1.31 ± 0.08 vs. 1.35 ± 0.06 in the first region of interest (ROI1) and ROI2 = 1.37 vs. 1.41. Hence they concluded that lower FD values in patients could indicate sub clinical erosive changes in the condyle, demonstrating the diagnostic value of fractal analysis. Further this was supported by Ozturk et al. [59], who through their CBCT based retrospective study proposed that fractal analysis might serve as a guide in identifying early pathological changes related to TMJ and by Cosgunarslan et al. [60], with their study on edentulous patients on CBCT. Similarly Gulec et al. [60], retrospectively analyzed panoramic radiographs of ankylosing spondylitis patients and reported lower FD values in affected individuals (1.38 ± 0.06) compared to controls (1.41 ± 0.07). Arsan et al. [60], conducted a prospective study comparing FD values between 100 TMD patients and 100 healthy controls using panoramic radiographs. Although the mean FD values were slightly lower in the TMD group (1.22 ± 0.06) compared to the control group (1.25 ± 0.06), the difference was not statistically significant (P > 0.05, ANOVA). However, the authors observed a general trend of decreasing FD with increased joint degeneration and suggested that fractal analysis may still be useful in improving diagnostic efficiency when combined with clinical examination parameters, such as mouth-opening measurements and joint sounds. Koprucu et al. [61], carried out a prospective MRI-based study assessed FD values in patients with unilateral disc perforation of the TMJ Table 5. The mean FD value in the affected joints was 1.07 ± 0.12, significantly lower than the healthy contralateral joints used as controls (1.20 ± 0.11; P = 0.001, independent t-test). The authors concluded that fractal analysis could serve as a predictive tool for TMJ disease by identifying internal disc derangement, effusion, and early degenerative changes, even in the absence of MRI findings.

**Table 5:** Studies using fractal analysis to monitor treatment and bone healing after surgery.

Author(s)	Year	Aim/Objective	Key Findings
Heo et al. [45]	2002	Evaluate FD for bone healing assessment post-orthognathic surgery	FD analysis provided better evaluation of healing than visual assessment
Park et al. [46]	2006	Assess FD changes in healing after orthognathic surgery	FD changes correlated with bone healing process
Koca et al. [47]	2010	Measure FD changes 18 months post-cyst surgery	Significant FD increase postoperatively, indicating bone regeneration
Kang et al. [48]	2012	Evaluate transient osteopenia after orthognathic surgery using FA	Found bone density reduction post-surgery due to regional acceleratory phenomenon
Akbulut et al. [51]	2020	Use FD on hand-wrist radiographs to guide rapid palatal expansion decisions	FD proved useful in predicting treatment success
Colak et al. [49]	2022	Observe FD changes in osteotomy lines and mandibular condyles after sagittal split osteotomy	FA revealed trabecular changes and supported its use for bone healing evaluation
Coban et al. [50]	2023	Compare FD changes post-genioplasty with or without mandibular advancement	No significant FD difference between groups; middle genial segment healed slower

### Fractal analysis of bone in MRONJ

Sahin et al. [62], compared panoramic radiographs of patients with early and advanced stages of medication-related osteonecrosis of the jaw (MRONJ). They found more significant bone structure alterations at advanced stages, though FD values did not vary significantly among the groups. Table 6 summarizes all the studies done using fractal analysis detection in bony changes in patients having systemic diseases.

**Table 6:** Studies using fractal analysis for detection in bony changes in patients having systemic diseases.

Author(s)	Year	Aim/Objective	Key Findings
Ergun et al. [52]	2008	Case report on HPT	FD improved post-parathyroidectomy
Arsan et al. [81]	2017	FD in TMJ disorders	Lower FD in more severe degeneration
Sahin et al. [62]	2019	FD in MRONJ stages	No significant FD difference overall; only certain ROIs differed
Gulec et al. [58]	2021	Evaluated FD in bruxism	Significant lower FD in condyles of bruxers
Kocak et al. [59]	2021	Condylar FD in malocclusion	FD lower in controls; FA useful in structural assessment
Turkmenoglu et al. [54]	2022	FD in Rheumatoid Arthritis patients	FD lower in condyles of RA patients; useful diagnostic tool
Gunacar et al. [82]	2022	Mandibular changes in psoriasis	FD and morphometric indices useful
Canger et al. [56]	2023	FD in ankylosing spondylitis	Lower FD in AS patients; risk of secondary osteoporosis
Koprucu et al. [61]	2023	MRI vs FD in TMJ perforation	FD varied with MRI subclassifications
Ozturk et al. [53]	2024	Bone changes in thyroid disorders	Lower FD in hyperthyroid; FA detects bone mass changes early
Balkan et al. [55]	2024	FD change post-BTX in bruxism	FD decreased in masseter muscle attachment area
Temur et al. [83]	2024	Jaw changes in children with RHD	FD showed unilateral and bilateral bone metabolism differences

#### Limitations of fractal analysis

Fractal analysis, although extensively researched, is not yet clinically applicable due to the following drawbacks,

1. Limited Reproducibility: The box-counting method remains the most widely applied FD algorithm, but inconsistencies across ROI selection, imaging modalities, and FD computation software tools and fractal algorithms (e.g., box-counting, power spectrum) may produce inconsistent results, thereby complicating comparisons.
2. Image Quality Dependence: Fractal analysis exhibits a significant sensitivity to image resolution, noise, and contrast. Variations in the quality of radiographs can markedly impact the precision of the findings [63, 64].
3. Standardization Challenges: The absence of uniform imaging protocols (e.g., exposure parameters, positioning) among various dental radiographs may result in variable fractal dimension measurements [10, 65].
4. Two-Dimensional Constraint: The majority of dental radiographs are two-dimensional, thereby restricting the evaluation of intricate three-dimensional bone structures, which may result in incomplete or deceptive interpretations [66].
5. ROI Selection Bias: The deliberate selection of the Region of Interest (ROI) introduces subjectivity and variability, which may compromise reproducibility and reliability [65].
6. Restricted Clinical Correlation & Applicability: Fractal dimensions may not consistently exhibit a direct relationship with clinical variables including bone density, disease severity, or therapeutic outcomes. Although FD is significantly able to describe diseased bone, the biological significance of alterations in fractal dimension is often ambiguous, which complicates clinical interpretation [67].
7. Impact of Anatomical Overlap: The presence of overlapping anatomical structures, such as trabeculae, roots, and sinus walls, may confound the accurate interpretation of trabecular patterns, thereby diminishing the precision of analyses [10, 65].

#### Clinical Applications and Integration of Fractal Analysis with Artificial Intelligence & Future Scope

- Artificial intelligence (AI)—particularly deep learning—has excelled in segmentation, detection, and classification of dental radiographic features which can be combined with the numerical bone texture metrics determined by fractal analysis. A recent overview of systematic reviews of studies by Turosz N et al. [68], using AI on panoramic radiographs found that there was a human-level (or better) performance in detecting periapical radiolucencies, missing teeth, caries, and other findings—processing thousands of images swiftly and accurately. Meanwhile, these reviews also highlighted the growing use of convolutional neural networks for tasks like segmentation and pathology detection in dental imaging, laying the groundwork for combining FD metrics with AI feature extraction to enhance diagnostic precision. Future research directions could be carried out:
- **Hybrid Intraoral Scanning Systems**: AI algorithms could automatically extract FD values from precisely identified regions of interest (ROIs), enabling real-time assessment of bone health, periodontal risk, and implant integration.
- **Multimodal Imaging**: Integration of FD analysis with CBCT or higher-resolution modalities could yield more robust bone-quality biomarkers across diverse patient populations.
- **Predictive Analytics**: Longitudinal and large-scale AI models incorporating FD trends could potentially forecast progression of early degenerative changes in TMJ, post extraction healing, post-surgical healing, and bone-related diseases, response to treatment, or implant outcomes.

## Conclusion

The integration of radiographic imaging with mathematic based pattern recognition in Fractal Analysis (FA) presents a new avenue for analyzing dental images. By measuring intricate bone patterns with dental X-rays, it provides an objective means of studying bone structure that traditional methods cannot capture. FA is non-invasive and cost-effective, helping diagnose conditions like osteoporosis and periodontitis by detecting early changes in bone structure that are not visible with standard imaging. It has shown sensitivity to changes after dental treatments. FA can also assess dental implants and monitor bony changes in various systemic diseases. Its applications are highly promising but require more standardized studies before it can be validated for use in everyday dental practice.

## Data Availability

Not applicable.
